# Editorial: Innovative immunotherapy strategies for enhanced treatment of Hodgkin and non-Hodgkin lymphomas

**DOI:** 10.3389/fimmu.2025.1642505

**Published:** 2025-06-20

**Authors:** Michael J. Robertson, Eric Vivier, Penny Fang

**Affiliations:** ^1^ Indiana University Melvin and Bren Simon Comprehensive Cancer Center, Indianapolis, IN, United States; ^2^ Centre d’Immunologie de Marseille-Luminy, Marseille, France; ^3^ Department of Radiation Oncology, The University of Texas MD Anderson Cancer Center, Houston, TX, United States

**Keywords:** lymphoma, immunotherapy, CAR (chimeric antigen receptor) T-cell therapy, immune checkpoint inhibitors, monoclonal Abs

Immunotherapy is a major component of current cancer treatment. Rituximab, one of the first efficacious immunotherapies for cancer, remains integral to treatment regimens for B cell lymphomas. Recent advances include approval of CD19-directed chimeric antigen receptor (CAR) T-cell therapies and CD20 x CD3 bispecific antibodies for relapsed and refractory B cell lymphomas (1, 2). Furthermore, immune checkpoint inhibitors (ICI) and CD30 antibody-drug conjugates have significantly improved treatment outcomes for classic Hodgkin lymphoma (cHL) (2). However clinical results remain suboptimal and further improvement in therapeutic options is needed. The seven original research articles, two case reports, and review article in this Research Project survey promising novel strategies for treatment of lymphoma.

Several articles investigate new approaches for CAR T-cell therapy. Efficacy of CAR T-cell therapy could be enhanced by use of CAR T-cells directed at more than one target antigen (3). Huang et al. and Luo et al. describe the safety and efficacy of dual CD20/CD30- and CD19/CD22-directed autologous CAR T-cells for treatment of aggressive B cell lymphomas. The patient in the report of Huang et al. had diffuse large B cell lymphoma (DLBCL) that had transformed from low-grade follicular lymphoma. The patient had durable complete response after CD20/CD30-directed CAR T-cell therapy even though the malignant cells expressed CD30 only weakly and partially. CD30 antibody-drug conjugate brentuximab vedotin has significant activity in DLBCL irrespective of CD30 expression (4). CD30 could be expressed by tumor cells at levels below the limit of detection by routine immunohistochemistry but sufficient to mediate antitumor activity. CD30-targeted agents could also affect tumor cells indirectly by modifying the tumor microenvironment. The patient did not experience cytokine release syndrome (CRS) or immune effector cell-associated neurotoxicity syndrome (ICANS).

Luo et al. took a sequential approach to treat a patient with refractory Burkitt lymphoma. High-dose chemotherapy and autologous peripheral blood stem cell transplantation was done to reduce tumor bulk and as lymphodepletion, followed by infusion of CD19/CD22-directed CAR T-cells. Maintenance treatment with anti-PD-1 antibody tislelizumab was given after the patient achieved complete response. In addition to employing a dual targeting extracellular domain, the third generation CAR used in this study had a novel intracellular signaling domain that included a CD3 zeta motif and costimulatory elements of CD28 and TLR2.

After apheresis has been done to collect autologous lymphocytes, bridging therapy is often needed to prevent rapid disease progression while awaiting production of CAR T-cells (5). Radiotherapy may offer advantages compared to chemotherapy for bridging therapy (5, 6). Ruan et al. have investigated the feasibility and potential mechanism of action of hyper-fractionated radiotherapy as bridging treatment prior to CD19-directed CAR T-cell therapy. Hyper-fractionated radiotherapy was safe and effective and, within limitations of an uncontrolled study, did not appear to affect incidence of CRS or ICANS. Exploratory studies suggested that bridging radiotherapy may have immunomodulatory effects.

Non-viral gene transfer techniques have been explored as an alternative to retroviral or lentiviral vectors for production of CAR T-cells. Transposon DNA plasmids (such as Sleeping Beauty and piggyBac) have significant advantages, including reduced production costs and immunogenicity compared to viral vectors, but are limited by lower transduction efficiency (7). Mucha et al. describe a novel procedure for non-viral production of CAR T-cells involving piggyBac transposons and irradiated allogeneic feeder cells. This approach allowed large scale production of clinical grade CD19-directed autologous CAR T-cells that were used successfully in clinical trials.

ICI have shown impressive activity for treatment of cHL and primary mediastinal large B cell lymphoma, but efficacy of ICI for most other types of non-Hodgkin lymphoma has been limited. Ricard et al. studied ICI-based treatment of angioimmunoblastic T-cell lymphoma (AITCL). The tumor microenvironment of AITCL often contains EBV-infected immunoblasts that promote overexpression of PD-L1 (8). Furthermore, mutations in genes regulating DNA methylation, including *TET2* and *DNMT3A*, are seen in peripheral T cell lymphomas, including AITCL (8, 9). Therefore Ricard et al. used hypomethylating agent 5-azacytidine together with ICI nivolumab to treat patients with AITCL. Treatment was well tolerated and overall response rate was 78%.

Ruggeri et al. examined expression of serine/threonine kinase CK2 in cHL. CK2a was overexpressed in cHL cell lines and Reed-Sternberg cells of patients with cHL. Silmitasertib, an inhibitor of CK2α, caused apoptosis of cHL cell lines and decreased expression of PD-L1. Thus CK2a may be a target for innovative therapies of cHL.

Rituximab, a type I chimeric CD20 monoclonal antibody (mAb) of IgG1 isotype, has revolutionized the treatment of B cell lymphomas. The mechanism of action of rituximab is complex and may involve antibody-dependent cellular phagocytosis (ADCP) by monocytes and macrophages, antibody-dependent cellular cytotoxicity by NK cells, and complement-dependent cytotoxicity. Nguyen et al. show that a CD20 mAb of IgG2 isotype (rituximab-IgG2) can enhance ADCP induced by different isotypes of rituximab and other therapeutic mAb. Rituximab-IgG2 augments ADCP in part by causing downregulation of CD47 on lymphoma cells. Rituximab-IgG2 also induced more apoptosis of lymphoma cells than rituximab-IgG1 or rituximab-IgG3.

Fan et al. investigated the causal relationship between metabolites, immune cell phenotypes, and risk of lymphoma and chronic lymphocytic leukemia (CLL) in Mendelian randomization analysis. Several metabolites and immune cell phenotypes were associated with different subtypes of lymphoma. However, evidence that immune cell phenotype was responsible for the effect of metabolites on risk of lymphoma was found only for DLBCL and CLL.

Wang et al. conducted a retrospective study of clinical characteristics of marginal zone lymphoma (MZL) and its response to treatment in the era of immunotherapy. MZL is a low-grade B cell lymphoma with three subtypes: extranodal MZL of mucosa-associated lymphoid tissue (MALT lymphoma), nodal MZL, and splenic MZL (10). Among 265 newly diagnosed patients with MZL reported by Wang et al., 66% had MALT lymphoma and the remaining cases were about equally distributed between nodal and splenic MZL. Overall response rates did not differ between patients receiving rituximab plus chemotherapy versus obinutuzumab plus chemotherapy. However, in a subgroup analysis of 51 patients with high tumor burden the overall response rate favored obinutuzumab over rituximab. This observation requires confirmation in prospective studies with larger numbers of patients that have high tumor burden.

Primary large B cell lymphomas of immune-privileged sites include primary DLBCL of the central nervous system (PCNSL), primary vitreoretinal DLBCL (PVRL), and primary testicular DLBCL (PTL). These aggressive lymphomas were grouped together in the 5^th^ Edition of the World Health Organization Classification of Lymphoid Neoplasms based on their common biological features (11). Wang et al. have reviewed the epidemiology, pathogenesis, prognosis, and therapy of PCNSL, PVRL, and PTL. They discuss several novel therapeutic approaches involving small molecule inhibitors, antibody-drug conjugates, bispecific antibodies, and CAR-T cells for treatment of these challenging diseases. Finally, one should also consider the development of Natural Killer cell engagers which are in clinical trial in non-Hodgkin B cell lymphomas (12).
